# UXT potentiates angiogenesis by attenuating Notch signaling

**DOI:** 10.1242/dev.112532

**Published:** 2015-02-15

**Authors:** Yi Zhou, Rui Ge, Rui Wang, Feng Liu, Yuefeng Huang, Heng Liu, Yan Hao, Qin Zhou, Chen Wang

**Affiliations:** 1 State Key Laboratory of Cell Biology, Institute of Biochemistry and Cell Biology, Shanghai Institutes for Biological Sciences, Chinese Academy of Sciences, Shanghai 200031, China; 2 State Key Laboratory of Biomembrane and Membrane Biotechnology, Institute of Zoology, Chinese Academy of Sciences, Beijing 100101, China; 3 The Division of Molecular Nephrology andthe Creative Training Center for Undergraduates, Chongqing Medical University, Chongqing 400016, China

**Keywords:** Notch signaling, Angiogenesis, UXT, Zebrafish, HUVECs

## Abstract

Angiogenesis is spatially and temporally orchestrated by a myriad of signaling pathways, including the Notch signaling pathway. Here, we identified UXT as an evolutionarily conserved and developmentally expressed protein, indispensable for intersegmental vessel (ISV) formation in zebrafish. Deficiency of UXT in zebrafish embryos results in shorter ISVs, loss of tip cell behavior, and impairment of endothelial cell migration and division. Significantly, UXT attenuates the expression of the Notch-responsive genes *in vitro* and *in vivo*. Mechanistically, UXT binds to the promoters of the Notch signaling target genes and specifically interacts with the transactivation region domain of the Notch intracellular domain (NICD), impairing the interaction between NICD and the transcription factor RBP-Jκ endogenously. This prevents RBP-Jκ/CSL from activation and thus inhibits the consequent gene inductions. Furthermore, blockade of Notch signaling rescues the angiogenesis defect caused by UXT knockdown both *in vitro* and *in vivo*. Taken together, the data presented in this study characterize UXT as a novel repressor of Notch signaling, shedding new light on the molecular regulation of angiogenesis.

## Abstract (precis)

**Summary**: UXT/ART27 is a novel repressor of Notch signaling that impairs the interaction between NICD and RBP-Jκ/CSL and is indispensable for zebrafish intersegmental vessel formation.

## INTRODUCTION

The blood vessel network is composed of arteries, capillaries and veins. Vasculogenesis is a *de novo* assembly of the network, whereas angiogenesis is the coordinated growth of endothelial cells (ECs) from pre-existing vasculature (Risau, 1997; Risau and Flamme, 1995). Both processes are essential for tissue growth and organ function in development, physiology and disease (Phng and Gerhardt, 2009).

Angiogenic sprouting in zebrafish is one of the best-characterized examples of angiogenesis both morphologically and mechanically (Childs et al., 2002; Lawson and Weinstein, 2002; Siekmann and Lawson, 2007). This model centers on the interplay between tip cell and stalk cells (Eilken and Adams, 2010; Gerhardt et al., 2003; Siekmann and Lawson, 2007). Displaying a gene expression profile distinct from that of the stalk cell, the tip cell is the spearhead selected to lead the emerging sprout, and it is followed by the endothelial stalk cells (Phng and Gerhardt, 2009). Studies of mouse and zebrafish development have firmly established that vascular endothelial growth factor (VEGF) and Notch signaling pathways are fundamental for the specification of endothelial cells into tip and stalk cells (Potente et al., 2011).

Notch is a family of large single-pass type I transmembrane protein receptors, which includes Notch 1-4 in mammals (Artavanis-Tsakonas et al., 1999), and Notch1a, Notch1b, Notch2 and Notch3 in zebrafish (Lorent et al., 2004; Westin and Lardelli, 1997). Correspondingly, there are multiple ligands that selectively bind to different Notch receptors. Engaging a ligand to Notch causes receptor proteolysis (trans-interactions), which is catalyzed by a series of proteases, resulting in the release of the Notch intracellular domain (NICD) and its translocation into the nucleus. When Notch signaling is off, RBP-Jκ/CSL mediates transcriptional repression by recruiting transcriptional co-repressor proteins (Bray, 2006). After Notch binding to the ligand and receptor proteolysis, the nuclear NICD interacts with RBP-Jκ/CSL through a conserved WxP motif in the Notch Rbp-associated molecule (RAM) domain, thus triggering the ‘transcriptional switch’ to activate the gene expression (Tamura et al., 1995). Notch target genes include the helix-loop-helix (HLH) family transcription factors (such as *hey1*, *her6*), which are important for regulating angiogenesis (Phng and Gerhardt, 2009). In zebrafish, Notch signaling potently suppresses vessel sprouting and represents an elegant model for studying cell fate determination in angiogenesis (Artavanis-Tsakonas et al., 1999; Siekmann and Lawson, 2007). We are intrigued to explore how the strength and duration of Notch signaling are modulated in the context of angiogenesis; in particular, we are interested in the molecular regulation of NICD in the nucleus.

The ubiquitously expressed transcript UXT (∼18 kDa, also known as ART27) was first identified in human and mouse (Markus et al., 2002; Schroer et al., 1999). UXT expression is markedly elevated in some human tumors (Zhao et al., 2005). Its functional characterization is largely unknown. We recently reported that UXT modulates innate immunity and inflammation, using *in vitro* cell models (Huang et al., 2011a,b; Sun et al., 2007). Interestingly, a global knockout of UXT in mouse is embryonic lethal (Y.Z., unpublished data), suggesting that UXT might play an important role during development. In this study, we characterize the UXT protein as a novel repressor of Notch signaling in angiogenesis, which functions by impairing the interaction between NICD and RBP-Jκ/CSL.

## RESULTS

### UXT is evolutionarily conserved and is expressed during development

**Fig. 1.**
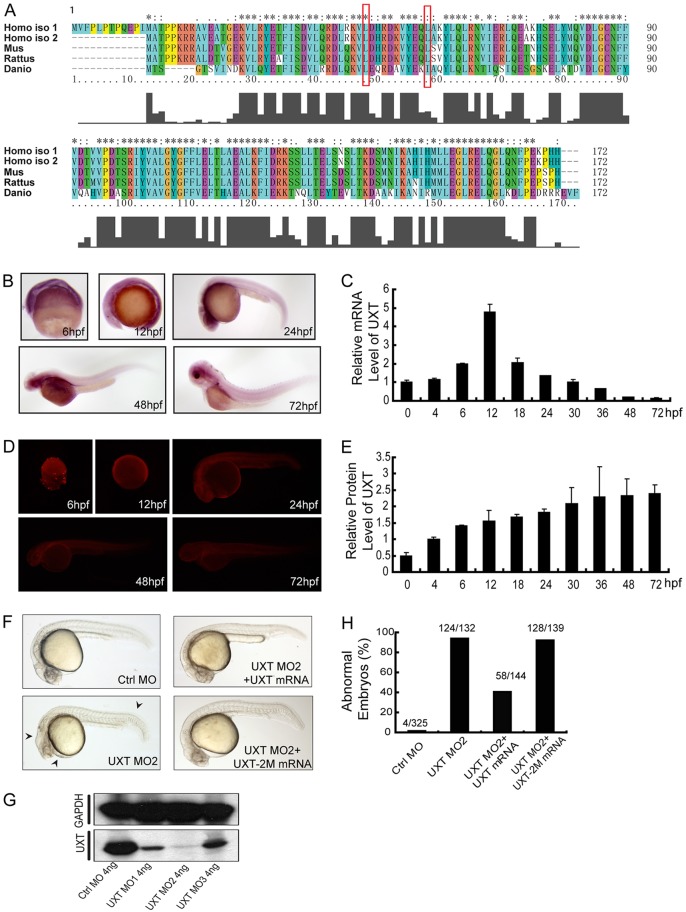
**UXT is evolutionarily conserved and developmentally expressed.** (A) A sequence alignment of human, mouse, rat and zebrafish UXT proteins by Clustal X. The histogram below the ruler indicates the degree of similarity. Peaks indicate positions of high similarity and valleys indicate low similarity. The asterisk indicates positions that have been fully conserved. The red rectangles indicate the conserved amino acid sites for UXT-2M mutation. (B) Whole-mount *in situ* hybridization of UXT in zebrafish embryos from 6 hpf to 72 hpf. The developmental time points are indicated in the panels. (C) Quantification of the UXT mRNA expression in zebrafish embryos from 6 hpf to 72 hpf by using RT-qPCR. Data show the mean±s.e.m. (at least three independent experiments). (D) Antibody staining of UXT in zebrafish embryos from 6 hpf to 72 hpf. (E) Quantification of UXT protein expression in zebrafish embryos with the UXT-specific antibody 4B4, from 0 hpf to 72 hpf, normalized to GAPDH. Densitometry was performed using ImageJ. Data show the mean±s.e.m. (at least three independent experiments). (F) Phenotypic analyses of zebrafish embryos at 24 hpf. Embryos were injected with 4 ng of control morpholino (Ctrl MO), 4 ng of UXT MO2 or 4 ng of UXT MO2 with 150 pg of UXT mRNA (UXT MO2+UXT mRNA). Additional embryos were injected with 4 ng of UXT MO2 plus 150 pg of UXT mRNA mutant (UXT MO2+UXT-2M mRNA). All embryos were analyzed at 24 hpf. The morphological defects are indicated by black arrowheads. (G) The knockdown efficiency of the morpholinos. Embryos were injected with 4 ng of control morpholino, 4 ng of UXT MO1, 4 ng of UXT MO2 or 4 ng of UXT MO3. The embryos were harvested at 24 hpf and the lysates were probed with the anti-zebrafish UXT-specific antibody 4B4. (H) Graphical representation of zebrafish phenotypic analyses; the number of embryos analyzed in each group is indicated above the bars.

Our bioinformatics analysis reveals that UXT is highly conserved across vertebrate species (Fig. 1A). The zebrafish UXT displays 50.6% sequence homology and 87.8% sequence similarities to human sequences (Fig. 1A). Using the whole-mount *in situ* hybridization (WISH) technique, we observed a ubiquitous expression of *uxt* mRNA as early as 6 hours post fertilization (hpf) in zebrafish embryos, and later on we saw a relative enrichment in the head region and parts of the trunk (Fig. 1B). The real-time quantitative PCR (RT-qPCR) results further substantiated the expression of *uxt* mRNA during zebrafish embryo development (Fig. 1C). A mouse monoclonal antibody against the zebrafish UXT protein (4B4) was generated and its specificity was confirmed (ABmart, Shanghai, China). Consistently, the protein expression of UXT mirrors that of the mRNA (Fig. 1D), but with a relatively stable expression level (Fig. 1E; supplementary material Fig. S3). In addition, we screened out three anti-sense morpholino oligonucleotides (MO1, MO2 and MO3, listed in supplementary material Table S1) that effectively knocked down UXT expression (Fig. 1G). A scramble morpholino sequence was included as a negative control (Ctrl MO). We observed that MO2 was the most efficient, and therefore it was used in the following experiments (Fig. 1G; supplementary material Figs S1 and S2). MO1 and MO3 were also used in some experiments (supplementary material Figs S4, S5, S7 and S10).

Depletion of UXT by injection of morpholino oligonucleotides caused a marked developmental abnormality, which was evidenced by the shortened trunk, curly tail, decreased pigmentation and hindbrain atrophy (Fig. 1F; supplementary material Fig. S4). UXT is an α-class prefoldin family protein, and its structure was predicted to contain two α-helix domains (Vainberg et al., 1998). By mutating two conserved leucine residues to proline residues (L45P/I54P in zebrafish; L50/59P in human) (Fig. 1A, red outline), we generated a loss-of-function mutant of UXT, UXT-2M. Interestingly, the developmental abnormalities were rescued by re-expression of wild-type *uxt* mRNA, but not by the UXT-2M mRNA (Fig. 1F,H). Taken together, these data suggest that UXT might play some role during zebrafish development.

### UXT modulates angiogenesis in zebrafish

To address the potential function of UXT in zebrafish development, we carried out microarray assays and compared relative mRNA expression levels between UXT-deficient and control embryos (72 hpf). Notably, the expression of genes associated with both Notch signaling (such as *dla*, *dlb*, *dld*) and angiogenesis (such as *birc5a*, *igfbp2*, *vegfaa*) were affected by UXT knockdown (data not shown).

**Fig. 2.**
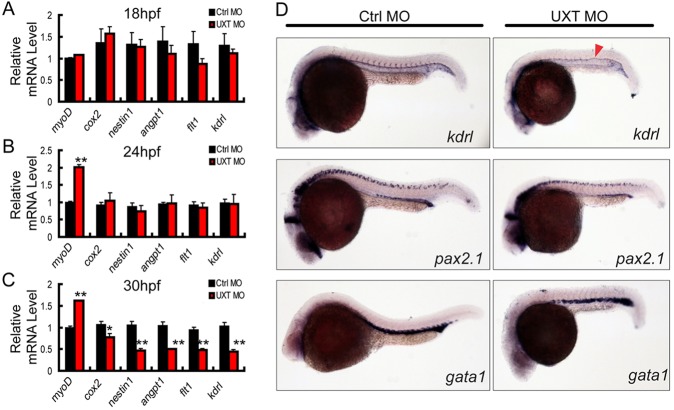
**UXT is essential for zebrafish angiogenesis.** (A-C) RT-PCR verification of angiogenesis markers in 18 hpf, 24 hpf or 30 hpf zebrafish embryos injected with 4 ng of control morpholino (Ctrl MO) or with 4 ng of UXT morpholino (UXT MO). Data shown are the mean±s.e.m. (at least three independent experiments); *P<0.05, **P<0.01 versus the corresponding control. (D) Whole-mount *in situ* hybridization of embryos injected with 4 ng of control morpholino (Ctrl MO) or 4 ng of UXT morpholino (UXT MO). Riboprobes against the angiogenesis marker *kdrl*, the pronephric marker *pax2.1* and the erythroid marker *gata1* were visualized at 22 hpf. The red arrowhead indicates the decreased expression of *kdrl* in UXT-deficient embryos.

It has been well established that the angiogenesis of intersegmental vessels (ISVs) mainly takes place at the time window from 18 hpf to 30 hpf in zebrafish (Fouquet et al., 1997). This led us to speculate that UXT might play a role in ISV development. To explore this possibility, we investigated mRNA expression of the angiogenesis markers, including *cox2*, *nestin1*, *angpt1*, *flt1* and *kdrl* (Albini et al., 1996; Amoh et al., 2005; Kim et al., 2006; Luttun et al., 2002; Stefater et al., 2011; Wu et al., 2006), and of the somite marker myod, at 18 hpf, 24 hpf and 30 hpf. When the expression of UXT was knocked down, these angiogenesis markers were expressed at slightly lower levels at 18 hpf and 24 hpf (Fig. 2A,B), but were strikingly decreased at 30 hpf (Fig. 2C; supplementary material Fig. S5).

Next, we examined the expression of the angiogenesis marker *kdrl* as well as additional lineage markers by using WISH. At 22 hpf, the expression of *kdrl* mRNA in UXT-deficient zebrafish embryos was markedly reduced in ISVs, whereas the pronephric marker *pax2.1* and the erythroid marker *gata1* were unaffected in UXT-deficient embryos (Fig. 2D). Taken together, these data demonstrate the importance of UXT on the process of zebrafish angiogenesis.

### UXT is crucial for endothelial cell fate determination in sprouting ISVs

**Fig. 3.**
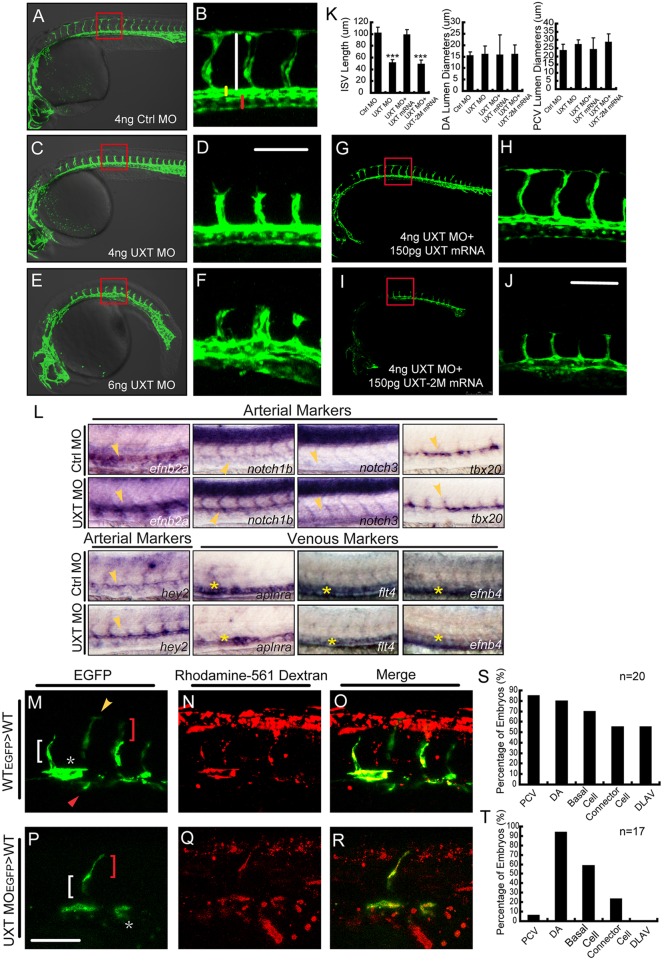
**UXT is indispensable for zebrafish ISV spouting.** (A-F) Confocal imaging of the UXT-deficient *Tg(kdrl:EGFP)^s843^* embryos at 30 hpf. (A,C,E) Bright-field images merged with confocal images. (B,D,F) Higher magnification images of the confocal-imaged area in the red boxed regions in A,C and E. (A) General morphology and trunk vascular phenotype and (B) the ISV sprouts in a *Tg(kdrl:EGFP)^s843^* embryo injected with 4 ng of control morpholino (Ctrl MO). (C) General morphology and trunk vascular phenotype, and (D) the ISV sprouts in a *Tg(kdrl:EGFP)^s843^* embryo injected with 4 ng of UXT morpholino (UXT MO). (E) General morphology and trunk vascular phenotype, and (F) the ISV sprouts in a *Tg(kdrl:EGFP)^s843^* embryo injected with 6 ng of UXT MO. The ISVs are marked by the vertical white line. The yellow and red bars indicate the lumen of the dorsal aorta (DA) and posterior cardinal vein (PCV), respectively. All of the embryos were confocal imaged at 30 hpf and are shown in lateral views with rostral left and dorsal up. Scale bar:100 µm. (G-J) Confocal imaging of the *Tg(kdrl:EGFP)^s843^* morphants rescued by UXT mRNA. (H,J) The confocal imaged area of the red boxed regions in G and I. (G) The trunk vascular phenotype and (H) the ISV sprouts in a *Tg(kdrl:EGFP)^s843^* embryo injected with 4 ng of UXT morpholino plus 150 pg of wild-type UXT mRNA (4 ng UXT MO+150 pg UXT mRNA). (I) The trunk vascular phenotype and (J) the ISV sprouts in a *Tg(kdrl:EGFP)^s843^* embryo injected with 4 ng of UXT MO plus mutant UXT mRNA (4 ng UXT MO+150 pg UXT-2M mRNA). All of the embryos were confocal imaged at 30 hpf. Scale bar: 100 µm. (K) Statistical analysis of the ISV length and the lumen diameter of the DA or PCV from embryos injected with Ctrl MO, UXT MO, UXT MO+UXT mRNA or UXT MO+UXT-2M mRNA. For one experiment, six embryos of each treatment were analyzed at 30 hpf. Data show the mean±s.e.m. (at least three independent experiments); ***P<0.001 versus the corresponding control. (L) The whole-mount *in situ* hybridization of UXT-deficient embryos at 30 hpf. Riboprobes are against the arterial markers *efn2a*, *notch1*, *notch3*, *tbx20*, *hey2* and the venous markers *aplnra*, *flt4* and *efnb4*. The yellow arrowheads denote the DA; the yellow asterisks indicate the PCV. (M-R) Confocal imaging of the donor *Tg(kdrl:EGFP)^s843^* cells in non-transgenic embryos at 30 hpf. (M) Wild-type cells contribute to all trunk vessels, including the DA (asterisk), PCV (red arrowhead), basal cells (red bracket), connector cells (white bracket) and DLAV (yellow arrowhead). (P) Deficient for UXT, the endothelial cells of *Tg(kdrl:EGFP)^s843^* can only contribute to theDA, basal cells and connector cells. (N,Q) The transplanted donor cells were stained with Rhodamine-561 dextran. (O,R) Merged images of EGFP and Rhodamine-561 dextran. Scale bar: 75 µm. (S,T) Quantification of wild-type (S) and UXT-deficient (T) donor *Tg(kdrl:EGFP)^s843^* cells in non-transgenic embryos. *n*, the number of successfully transplanted embryos.

To further dissect the function of UXT in angiogenesis, we analyzed the ISV development of *Tg(kdrl:EGFP)^s843^* transgenic zebrafish using confocal microscopy. As expected, ISVs of control embryos extended to the dorsal-most aspect of the neural tube at 30 hpf, and T-shape branches formed by dorsal longitudinal anastomotic vessels (DLAV) were normal (Fig. 3A,B). Remarkably, ISVs of the embryos that were injected with 4 ng of UXT MO sprouted to the horizontal myoseptum and did not reach the dorsal roof to form DLAVs (Fig. 3C,D). Furthermore, the embryos that were injected with 6 ng of UXT MO displayed severe defects, such as a shorter trunk and a more downward curly tail (Fig. 3E). Notably, the ISVs of UXT-deficient embryos projected up at the midline of the trunk, and almost no ISVs were generated at posterior part of the trunk (Fig. 3E,F).

The length of ISVs in UXT morphants was significantly shorter, approximately half that of the control ones (51.3±5.2 µm versus 101.8±9.6 µm, *P*<0.005; Fig. 3K). The lumen diameter of the dorsal aorta remained roughly the same for both UXT morphants and control embryos. However, the lumen of the posterior cardinal vein (PCV) became narrower in UXT morphant embryos than in control embryos (27.2±2.8 µm versus 23.6±3.4 µm, *P*<0.05; Fig. 3K). The defects in ISVs in UXT morphants were rescued by injection of the morpholino-resistant form of the full-length UXT mRNA (Fig. 3G,H,K), but were not rescued by injection of the mutant UXT-2M mRNA (Fig. 3I-K). These observations indicate that UXT plays an essential function in promoting angiogenesis.

To explore whether UXT affected the arterial-venous identity, we examined the expression of arterial and venous markers in 30 hpf zebrafish embryos. *efnb2a*, *notch1b*, *notch3*, *hey2* and *tbx20* are arterial markers that are selectively expressed in the dorsal aorta area, whereas venous markers, such as *aplnra* (also known as msr), *flt4* and *ephb4* are expressed exclusively in the PCV region (Fischer et al., 2004; Lawson et al., 2001; Saint-Geniez et al., 2003; Szeto et al., 2002; Thompson et al., 1998; Wang et al., 1998). The arterial markers *efnb2a*, *notch1b* and *hey2* were expressed at a much higher level in UXT-deficient embryos than in control embryos (Fig. 3L).

To explore the role of UXT in determining the ISV cell fate, we carried out cell transplantation experiments. We isolated the cells from *Tg(kdrl:EGFP)^s843^* zebrafish into which either control MO or UXT MOs were injected, and transplanted these cells into wild-type host embryos at the sphere stage. The donor cells from *Tg(kdrl:EGFP)^s843^* were also labeled with lineage tracer in order to mark the amount of donor-derived cells in host embryos. The contribution of donor cells to different trunk vascular cell types was examined. GFP-positive donor cells from *Tg(kdrl:EGFP)^s843^* control embryos appeared at 30 hpf in all types of cells located in the trunk blood vessels (Fig. 3M-O). We confirmed this observation in 20 successfully transplanted embryos (Fig. 3S). By contrast, donor cells from UXT-deficient *Tg(kdrl:EGFP)^s843^* embryos appeared predominantly in the dorsal aorta (16/17 embryos, Fig. 3P-R), but were barely seen in the PCV (1/17 embryos). In addition, these donor cells were also observed in basal cells (10/17 embryos) and connector cells (4/17 embryos). Strikingly, no UXT-deficient donor cells had populated the DLAV (0/17 embryos, Fig. 3T). These results indicate that UXT is required cell autonomously for ISV formation. We also noticed that the phenotypes of UXT-deficient embryos echoed those observed following the activation of Notch signaling as reported recently (Siekmann and Lawson, 2007).

### UXT is crucial for endothelial cell migration and division

**Fig. 4.**
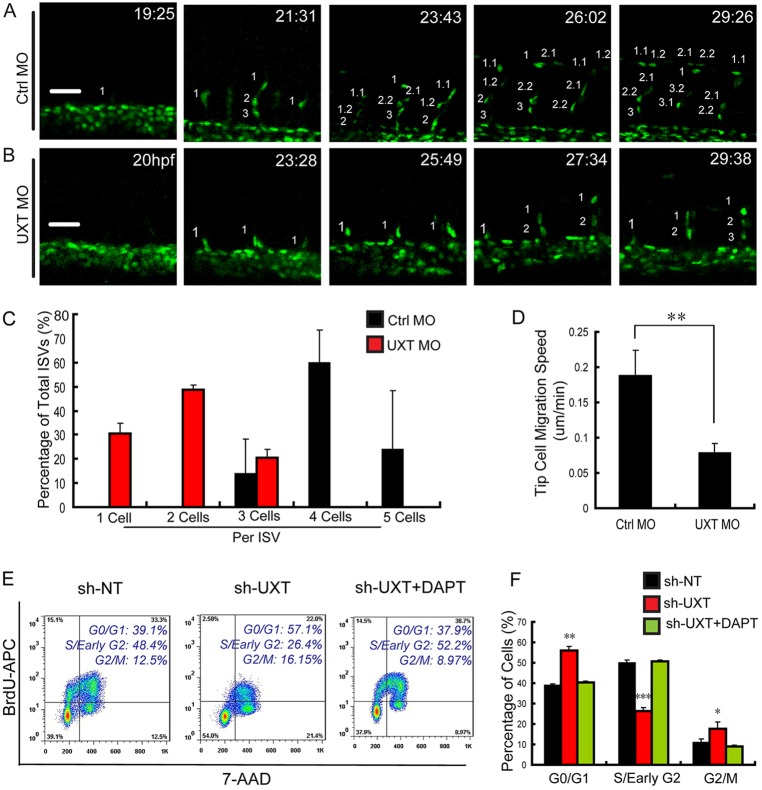
**UXT modulates endothelial cell migration and cell cycle.** (A,B) Confocal time-lapse images (from 19 hpf to 30 hpf) of *Tg(kdrl:EGFP)^s843^* embryos injected with 4 ng of control MO (A) or 4 ng of UXT MO (B). The numbering on the images shows the indicated time points. Scale bars: 50 µm. (C) Quantification of endothelial cell number in the segmental artery sprouts. (D) Quantification of the migration speed of ISV tip cells in control embryos and UXT morphants. (E,F) The inhibitory effect of UXT on cell cycle. (E) Flow cytometric analysis for BrdU incorporation and 7-AAD labeling in wild-type and UXT-deficient HUVECs or UXT-deficient HUVECs treated with 1.5 µM DAPT. (F) Quantification of the percentage of cells in different cell cycle stages. All quantitative data are the mean±s.e.m. (at least three independent experiments);*P<0.05, **P<0.01, ***P<0.001 versus the corresponding control.

To elucidate the cellular function of UXT, we further investigated ISV formation by *in vivo* time-lapse confocal microscopy, using *Tg(fli1:nEGFP)^y7^* transgenic fish. Tip cells appeared from the dorsal aorta at around 19 hpf, then they migrated to the horizontal myoseptum (Fig. 4A, 21:31 and 23:43). The majority of tip cells underwent cell divisions two or three times. Ultimately, tip cells migrated dorsally and formed the DLAV normally during ISV formation (Fig. 4A, 26:02 and 29:26). By contrast, tip cells in UXT-deficient *Tg(fli1:nEGFP)^y7^* embryos sprouted late, at ∼23 hpf (Fig. 4B, 20 hpf and 23:28), and then initiated cell divisions at ∼28 hpf (Fig. 4B, 27:34). Remarkably, these tip cells did not migrate further over the horizontal myoseptum (Fig. 4B, 29:38).

We noticed that most ISVs in control embryos consisted of three or four cells (Fig. 4C), whereas those in UXT-deficient embryos contained only one or two cells (Fig. 4C). The migration speed of UXT-deficient tip cells (0.079±0.012 µm/min) was much lower than that of control ones (0.188±0.036 µm/min, *P*<0.005; Fig. 4D).

Given that UXT-deficient embryos displayed severe defects in cell migration and division, we speculated that UXT might regulate the cell cycle of endothelial cells. To test this, the endogenous UXT in human umbilical vein endothelial cells (HUVECs) was knocked down by using short hairpin (sh)RNA, and the alteration of the cell cycle was measured (Fig. 4E). Consistently, UXT-deficient HUVECs displayed a marked increase in the number of G0-G1 phase cells, and a corresponding decrease in the number of cells in S phase (Fig. 4E,F), which strongly suggested that knockdown of UXT blocked cell cycle at G0-G1 phase. This cell cycle blockage was relieved by treatment with a Notch signaling inhibitor, DAPT (Fig. 4E,F). Taken together, these data establish the crucial function of UXT in endothelial cell migration and division.

### UXT attenuates Notch signaling by targeting NICD

**Fig. 5.**
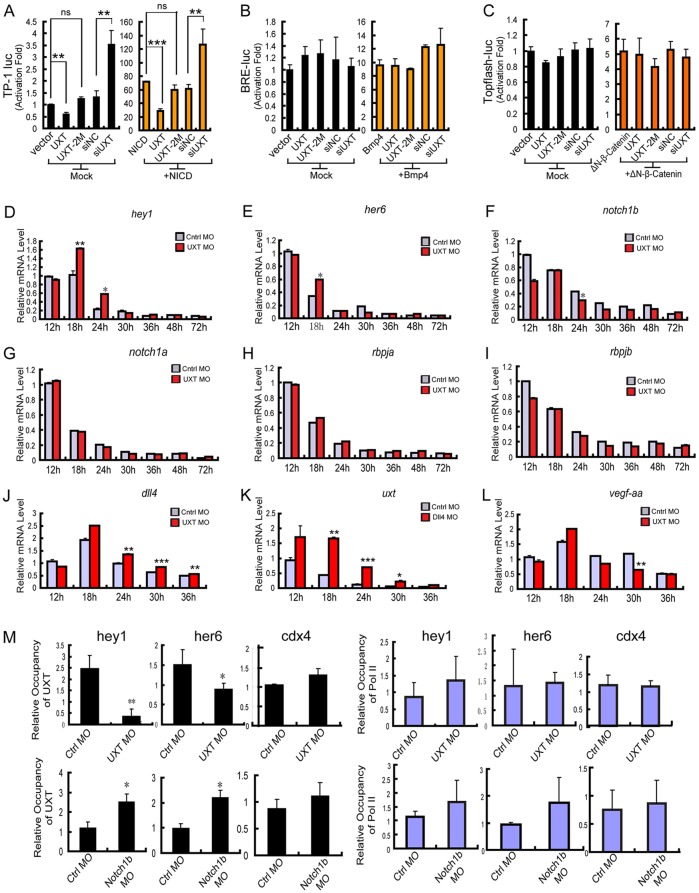
**UXT attenuates the expression of the Notch target genes.** (A) UXT impairs the TP-1 luciferase reporter of Notch signaling. The indicated plasmids and/or siRNAs were transfected into Cos-7 cells together with TP-1 reporter plasmids, in the presence or absence of NICD. (B) UXT does not influence BMP signaling. The indicated plasmids and/or siRNAs were transfected into Cos-7 cells together with the BRE reporter plasmids. After transfection, cells were starved for 12 h and then treated with or without BMP4 (10 ng). (C) UXT does not influence WNT signaling. The indicated plasmids and/or siRNAs were transfected into HEK293T cell lines together with the TOPflash reporter plasmids, in the presence or absence of ΔN-β-catenin. Data in A-C were normalized to empty vectors and are presented as the mean±s.e.m. (*n*=3). (D,E) UXT attenuates the expression of Notch target genes. The control and UXT-deficient zebrafish embryos (from 12 hpf to 72 hpf) were harvested, and the expression of *hey1* (D) and *her6* (E) were measured by RT-PCR. (F-J) UXT does not influence the expression of *notch*, *rbpja* or *rbpjb* but does affect *dll4*. The control and UXT-deficient zebrafish embryos (from 12 hpf to 72 hpf) were harvested, and the expression of *notch1a* (G), *notch1b* (F), *rbpja* (H), *rbpjb* (I) and *dll4* (J) were measured by RT-PCR. (K) DLL4 attenuates the expression of UXT. The control and DLL4-deficient zebrafish embryos (from 12 hpf to 36 hpf) were harvested, and the expression of *uxt* was measured by RT-PCR. (L) The relative mRNA expression level of *vegfaa* in control and UXT-deficient embryos (from 12 hpf to 36 hpf). Data shown in D-L are the mean±s.e.m. (at least three independent experiments). (M) E-ChIP assays at 24 hpf. Embryos injected with 4 ng of control MO, 4 ng of UXT MO or Notch1b MO were probed with the antibody against UXT and polymerase II, respectively. The data were normalized on the basis of the corresponding input control and are presented as the mean±s.e.m. (at least three independent experiments). For all panels, *P<0.05; **P<0.01; ***P<0.001; ns, non-significant versus the corresponding control or as indicated.

In light of the microarray analysis and the impact of UXT deficiency on Notch signaling activation, we reasoned that UXT might modulate the Notch signaling pathway. To explore this possibility, Notch-TP-1-luciferase reporter plasmids were introduced into UXT-deficient cells, in the presence or absence of NICD. Interestingly, knockdown of UXT potentiated the activation of the Notch-TP-1-luciferase reporter, whereas ectopic expression of UXT attenuated the same reporter expression (Fig. 5A). Notably, ectopic expression of UXT-2M displayed only a marginal effect on the expression of the Notch-TP-1-luciferase reporter (Fig. 5A), which was consistent with its inability to rescue the developmental abnormalities induced by the UXT morpholino (Fig. 1F). BMP signaling and Wnt signaling have been reported to be crucial signaling pathways modulating angiogenesis (Phng et al., 2009; Rothhammer et al., 2007). However, UXT did not affect the activation of BMP signaling or Wnt signaling, according to the results from a BRE reporter assay or a TOPflash reporter assay, respectively (Fig. 5B,C).

Next, we checked whether UXT could modulate Notch signaling *in vivo*. *her6* and *hey1* in zebrafish are homologous to *HES1* and *HEY1* in human, respectively. We observed that all of these genes were expressed at significantly higher levels at ∼18 hpf and 24 hpf when endogenous UXT expression was knocked down (Fig. 5D,E). We also evaluated other Notch target genes, such as *hey2* and *heyl*, and obtained similar results (supplementary material Figs S6 and S7). In addition, we observed no obvious alterations in the expression of the signaling proteins in the Notch pathway (including *notch1a*, *notch1b*, *rbpja* and *rbpjb*) in control embryos, but a slightly lower level of *notch1b* expression at 24 hpf in UXT-deficient embryos (Fig. 5F-I). Interestingly, the expression of *dll4* increased in UXT-deficient embryos (Fig. 5J), and reciprocally, the expression level of *uxt* was higher in *dll4*-deficient embryos (Fig. 5K). Consistently, *vegfaa* expression was downregulated in UXT-deficient embryos from 18 hpf to 30 hpf (Fig. 5L). These data indicate that UXT regulates Notch signaling per se.

To further address the action of UXT in Notch signaling, we performed a whole-embryo chromatin immunoprecipitation (E-ChIP) assay in 24 hpf zebrafish embryos, and the conserved promoter sequences of the RBP-Jκ binding site (5′-GTGGGAA-3′) (Ling et al., 1993) were analyzed. Consistently, UXT bound to the promoter regions of *hey1* and *her6*, but not to the promoter regions of the control gene *cdx4* (Fig. 5M). Interestingly, in *notch1b*-deficient embryos, an increase in UXT binding to the promoter regions of Notch target genes was observed (Fig. 5M).

**Fig. 6.**
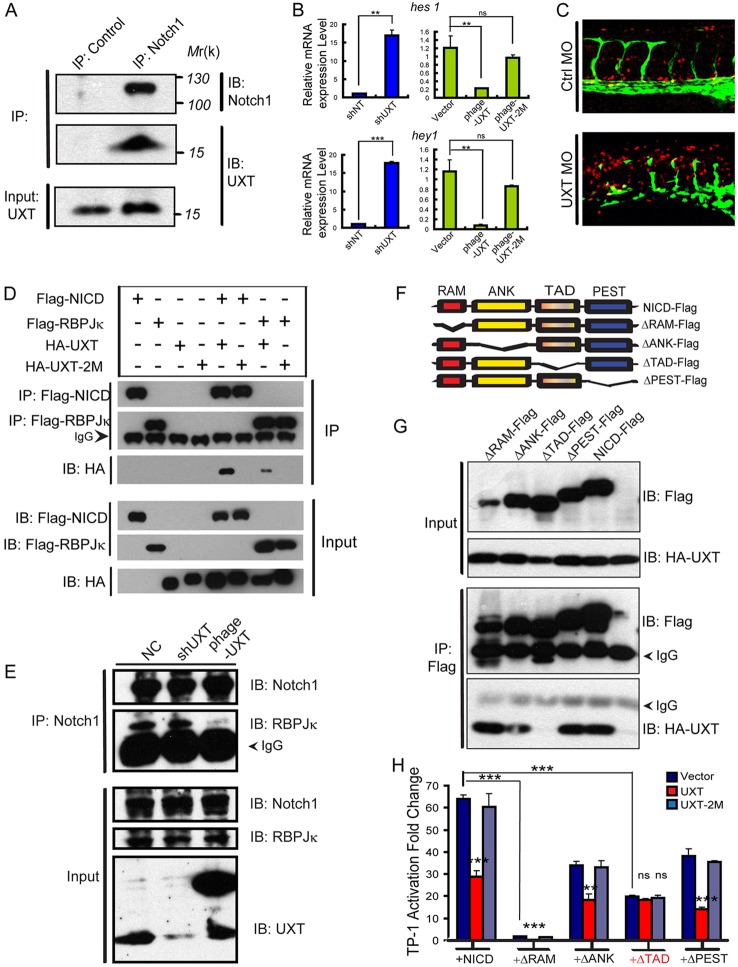
**UXT interacts with NICD endogenously through the Notch TAD domain.** (A) UXT interacts with NICD endogenously. HUVEC cell lysates were immunoprecipitated (IP) with an anti-Notch1 antibody or control IgG antibody. The immunoprecipitates were immunoblotted (IB) with the indicated antibodies. (B) UXT directly attenuates Notch-targeted genes in endothelial cells. The relative expression level of *hes1* and *hey1* were measured by RT-PCR. The data were normalized on the basis of the corresponding input control, and are presented as the mean±s.e.m. (at least three independent experiments). (C) UXT attenuates Notch signaling *in vivo*. Confocal images of *Tg(TP1:mCherry; fli1:EGFP)^y1^* fish injected with 4 ng of control MO or 4 ng of UXT MO. (D) UXT interacts specifically with NICD. HA-UXT or HA-UXT-2M was co-transfected with Flag-NICD or Flag-RBP-Jκ. Cell lysates were subjected to an immunoprecipitation assay using the anti-Flag antibody, followed by western blot analysis with anti-HA and anti-Flag antibodies. (E) UXT impairs the interaction between NICD and RBP-Jκ. HUVECs were treated with shUXT or phage-UXT-Flag. The endogenous NICD was immunoprecipitated with the anti-Notch1 antibody, and the immunoprecipitates were probed with the anti-RBP-Jκ antibody. (F) Schematic representation of the NICD deletion constructs used in the following experiments. (G) The TAD domain of NICD mediates its interaction with UXT. HA-UXT was co-transfected with ΔRAM-Flag, ΔANK-Flag, ΔTAD-Flag, ΔPEST-Flag or NICD-Flag. Cell lysates were subjected to an immunoprecipitation assay using an anti-Flag antibody. (H) UXT directly impaired Notch signaling. The indicated plasmids were transfected into Cos-7 cells together with TP-1 reporter plasmids, in the presence of NICD or the deletion mutants. Data are presented as the mean±s.e.m. (*n*=3); **P<0.01; ***P<0.001; ns, non-significant versus the corresponding control or as indicated.

Next, we explored whether UXT interacted with any components of the transcriptional protein complex. The endogenous UXT was pulled down by NICD in HUVECs (Fig. 6A), indicating that UXT interacted with NICD. In addition, UXT directly regulated the endogenous expression of Notch target genes, such as *hes1*, *hey1* (Fig. 6B), *hey2* and *heyl* (supplementary material Fig. S8). Knockdown of UXT enhanced the expression of Notch-responsive genes significantly, whereas the expression level of these genes was remarkably reduced by ectopic expression of UXT in HUVECs. To prove that UXT directly regulates Notch-responsive genes, we crossed the transgenic *Tg(TP1:mCherry)* fish with *Tg(fli1:EGFP)^y1^* fish, and observed a robust activation of the TP1 reporter in UXT-deficient embryos (Fig. 6C; supplementary material Fig. S10).

Next, HA-UXT was co-introduced with either Flag-NICD or Flag-RBP-Jκ into HEK293T cells. The co-immunoprecipitation experiments revealed that UXT strongly interacted with NICD, but interacted only marginally with RBP-Jκ (Fig. 6D). Consistently, UXT-2M failed to interact with either NICD or RBP-Jκ, indicating the functional relevance of the interaction between UXT and NICD.

In addition, knocking down or ectopically expressing UXT did not affect the stability of either Notch1 or RBP-Jκ. Notably, ectopically expressed UXT impaired the interaction between NICD and RBP-Jκ, as demonstrated by the endogenous immunoprecipitation experiment (Fig. 6E). Furthermore, UXT did not affect the cleavage or stability of the Notch1 receptor (supplementary material Figs S11 and S12).

To determine the mechanism by which UXT modulates Notch signaling activation, we generated several Flag-NICD truncation mutants (Fig. 6F). Each individual truncation mutant was co-expressed with HA-UXT in HEK293T cells, and then immunoprecipitated by using an anti-Flag antibody. We observed that HA-UXT was not pulled down by a transactivation region (TAD) domain deletion mutant of NICD (ΔTAD-Flag, Fig. 6G), indicating that the TAD domain of NICD mediates its interaction with UXT. To corroborate, deletion of the RAM or TAD domain of NICD significantly impaired the NICD-induced TP-1 activation (Fig. 6H), because the RAM domain mediates the interaction between NICD and RBP-Jκ. As expected, co-transfection of UXT with ΔTAD-NICD did not result in any further repression of Notch signaling activation. These data demonstrate that UXT directly interacts with the TAD domain of NICD, thus serving as a negative regulator of the Notch signaling.

### UXT facilitates angiogenesis by repressing Notch signaling

**Fig. 7.**
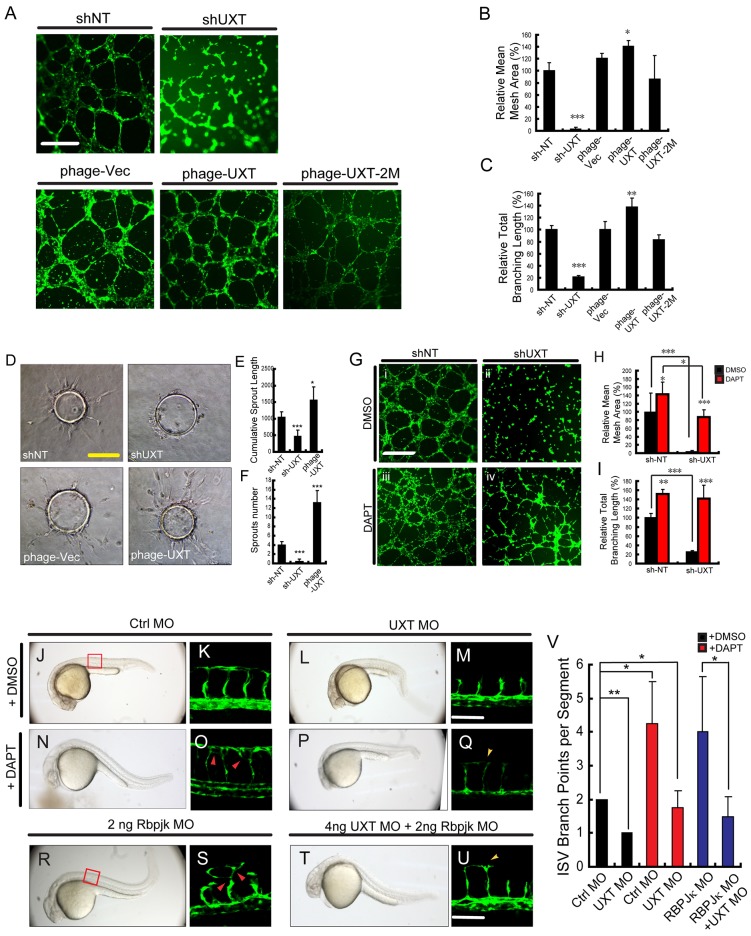
**See next page for figure legend.** **UXT potentiates angiogenesis** **through** **Notch signaling.** (A-C) UXT potentiates angiogenesis *in vitro*. (A) Knocking down UXT by shRNAs or ectopically expressing wild-type or mutant UXT (phage plasmids) was performed in HUVECs, followed by the *in vitro* angiogenesis assay. Fluorescence images were taken (A) and quantified (B,C). Scale bar: 500 µm. Relative mean mesh area (B) and relative branching length (C) were calculated by using Image J. (D-F) 3D *in vitro* angiogenesis with collagen gel-embedded cytodex beads coated with HUVECs, treated with shNT, shUXT, phage-vector or phage-UXT. Cumulative length and the numbers of all sprouts originating from an individual cytodex bead were quantified using ImageJ after 24 h, with ten cytodex beads analyzed per experimental group. Scale bar: 200 µm. (G-I) Fluorescence images (G) and quantification (H,I) of the *in vitro* angiogenesis assay on HUVECs treated with DMSO or DAPT. Wild-type (shNT) or UXT-deficient (shUXT) HUVECs were seeded on Matrigel and then treated with either DMSO or DAPT (1.5 µM) for 6 h. Scale bar: 500 µm. Relative mean mesh area (H) and relative branching length (I) were calculated using ImageJ. The data were normalized on the basis of the corresponding input control. (J-Q) *Tg(kdrl:EGFP)^s843^* embryos were treated with either 60 µM DAPT or DMSO. (J,K) *Tg(kdrl:EGFP)^s843^* embryos injected with 4 ng of control morpholino (Ctrl MO) were treated with DMSO. (N,O) *Tg(kdrl:EGFP)^s843^* embryos injected with 4 ng of control morpholino were treated with DAPT. (L,M) *Tg(kdrl:EGFP)^s843^* embryos injected with 4 ng of UXT morpholino (UXT MO) were treated with DMSO. (P,Q) *Tg(kdrl:EGFP)^s843^* embryos injected with 4 ng of UXT morpholino were treated with DAPT. All images were taken at 30 hpf. The confocal images in K,O,M,Q show higher magnification of areas of the bright-field images to the left. The red arrowheads indicate the robust angiogenesis in ISVs, whereas the yellow arrowheads highlight the DLAV growth rescued by DAPT treatment. Scale bar: 100 µm. (R-U) 2 ng of RBP-Jκ morpholino were injected into *Tg(kdrl:EGFP)^s843^* embryos without (R,S) or with (T,U) 4 ng of UXT morpholino. All the images were taken at 30 hpf. The confocal images in S,U show higher magnification of areas of the bright-field images to the left. Red arrowheads indicate excessive angiogenesis in ISVs, and yellow arrowheads denote DLAV growth rescued by co-injection of the RBP-Jκ morpholino. Scale bar: 100 µm. (V) Quantification of J-U. The ISV branching points per segment were analyzed. Five different embryos were analyzed per experimental group. The data were normalized on the basis of the corresponding input control. All quantitative data are presented as the mean±s.e.m. (at least three independent experiments); *P<0.05, **P<0.01, ***P<0.001 versus the corresponding control.

To substantiate whether UXT modulates angiogenesis by targeting Notch signaling, an *in vitro* angiogenesis assay was employed. HUVECs were seeded on Matrigel and they migrated to establish capillary-like structures with a lumen. As expected, control HUVECs formed normal capillary-like structures (Fig. 7A, shNT and phage-vec). By contrast, UXT-deficient HUVECs became rounded as isolated cells and failed to establish contacts with adjacent cells (Fig. 7A, sh-UXT). When HUVECs ectopically expressed UXT (Fig. 7A, phage-UXT), these cells formed more-robust capillaries and cord-like structures (Fig. 7A, phage-UXT). Quantitatively, ectopic expression of UXT induced a 1.5-fold increase in the mean mesh area (Fig. 7B) and branching length (Fig. 7C), as compared with the control HUVECs. Consistently, ectopic expression of UXT-2M caused a marginal dominant-negative effect on the angiogenesis (Fig. 7A, phage-UXT-2M; Fig. 7B,C).

We further employed a three-dimensional (3D) angiogenesis assay based on cytodex microcarriers. UXT was either knocked down or overexpressed in HUVECs, and cytodex beads were coated with these cells and then embedded into collagen gels. The outgrowth of capillary-like structures was assessed. Consistent with the reduced tube formation in the Matrigel assay, silencing of UXT expression caused the loss of sprouting activity. By contrast, ectopic expression of UXT markedly potentiated the sprouting from cytodex beads (Fig. 7D-F).

Furthermore, we treated HUVECs with DAPT or DMSO. DAPT is a γ-secretase inhibitor that prevents the S3 cleavage of Notch and mimics the inhibition of Notch signaling. Notably, DAPT treatment reversed the defects caused by knocking down endogenous UXT (Fig. 7Gii,iv), which was substantiated by quantifying the increase in the mesh area (Fig. 7H) and total branching length (Fig. 7I) during *in vitro* angiogenesis.

Finally, to substantiate the function of UXT in modulating Notch signaling *in vivo*, we treated *Tg(kdrl:EGFP)^s843^* embryos with 60 µM DAPT or DMSO at 12 hpf, and then observed ISV formation by confocal microscopy at 30 hpf. For the control embryos, DAPT treatment led to morphological defects of prolonged trunks and upward curly tails (Fig. 7N). Notably, ISVs displayed excessive growth (Fig. 7O). For UXT morphants, DAPT treatment partially rectified abnormal morphology, resulting in approximately normal trunks (Fig. 7P) and ISV growth (Fig. 7Q,V). In addition, DAPT treatment relieved the cells from the cell cycle blockage in UXT-deficient HUVECs (Fig. 4E,F). Consistently, injection of the RBP-Jκ morpholino caused the robust growth of ISVs, which phenocopied the results of DAPT treatment on embryos (Fig. 7R,S). Interestingly, co-injection with RBP-Jκ morpholino partially rescued the ISV growth defects in UXT-deficient embryos (Fig. 7T-V). Collectively, these data establish that UXT potentiates angiogenesis by attenuating Notch signaling per se.

## DISCUSSION

Angiogenesis is the growth of new blood vessels from pre-existing ones. It is a major challenge to identify and characterize pro- and/or anti-angiogenesis signals, and to elucidate how these signals influence the corresponding cellular responses in blood vessel formation. In particular, it remains elusive how the relevant signaling is modulated temporally and spatially in developmental and tumor angiogenesis. In this study, we have characterized UXT (also known as ART27) as a novel modulator that potentiates angiogenesis *in vivo* and *in vitro*, by inhibiting Notch signaling.

The sprouting of ISVs in zebrafish is a well-established model for studying angiogenesis. Notch and VEGF signaling play major roles in the vascular cell fate determination (Phng and Gerhardt, 2009; Williams et al., 2006). Tip cells are characterized by the expression of higher levels of mRNA encoding DLL4 and VEGFR2 (also known as KDRL – ZFIN), whereas stalk cells are rich in Notch and VEGFR1 (also known as FLT1 – ZFIN). In this study, we observed that the mRNA expression level of both *flt1* and *kdrl* were significantly lower in UXT-deficient embryos at 22 hpf, but the expression of *vegfaa* apparently did not change at that time (Fig. 5L). In addition, the expression of *notch1b* was attenuated in UXT-deficient embryos as early as 12 hpf (Fig. 5F), whereas the expression of *hey1* and *her6* was increased in UXT-deficient embryos from 18 hpf to 24 hpf. Interestingly, we did observe the alteration of *vegfaa* expression after 48 hpf in UXT-deficient embryos. It has been reported that VEGF induces DLL4 (Lobov et al., 2007), whereas Notch selectively inhibits VEGFR expression (Williams et al., 2006). Interestingly, we observed the reduced expression of *kdrl* in UXT-deficient embryos at 22 hpf. The expression of *vegfaa* but not *vegfab* (supplementary material Fig. S9) was decreased during the time window between 18 hpf to 30 hpf, whereas the expression of Notch target genes and *dll4* was markedly enhanced. The cell-autonomous role of Notch and VEGF signaling needs to be further addressed in future studies. It remains to be explored how UXT modulates VEGF signaling indirectly.

UXT interacts with NICD endogenously. Ectopic expression of wild-type UXT markedly inhibited the expression of Notch target genes, whereas ectopic expression of UXT-2M (L50/59P) failed to do so. Notch1-NICD contains a RAM (RBP-Jκ-associated-molecule) domain, an Ankyrin (ANK) domain with seven repeats, a TAD domain and a C-terminal PEST sequence (Choi et al., 2011). We identified that UXT interacts mainly with the TAD domain of NICD. Consistently, when using this TAD truncation mutant as the stimulus, UXT failed to repress the Notch-dependent activation, indicating that UXT potentiates angiogenesis by impairing Notch signaling. This is consistent with the reports that Notch signaling attenuates ISV development.

RBP-Jκ represses Notch target genes by recruiting transcriptional co-repressors, which include SMRT, SHARP, SKIP and CIR in mammals (Hsieh et al., 1999; Kao et al., 1998; Zhou et al., 2000). SMRT and SHARP directly interact with RBP-Jκ, whereas SKIP binds both SMRT and NICD. Unexpectedly, our E-ChIP assays revealed that UXT could constitutively bind the promoter region of Notch target genes. Furthermore, UXT predominantly interacted with NICD, but interacted only marginally with RBP-Jκ. Because DAPT directly reduced the endogenous NICD level, this treatment could rescue the angiogenesis defects caused by deficiency of UXT *in vitro* or *in vivo*. Knockdown of RBP-Jκ could also achieve the similar effects. Thus, we propose that UXT is another basal co-repressor of Notch target genes. Once NICD enters the nucleus, UXT is recruited onto the promoter sequences of Notch target genes. UXT interacts with the TAD domain of NICD and competes NICD away from RBP-Jκ, thus potently inhibiting the induction of Notch target genes. Future biophysical studies will hopefully provide mechanistic details for this hypothesis.

Angiogenesis is an indispensable vehicle for tumor metastasis, allowing oxygen and nutrients to be replenished and cancer cells to be mobilized (Carmeliet and Jain, 2011). Notch mutations were found in ∼40-70% of T-cell acute lymphoblastic leukemias, and in 10-15% of chronic lymphocytic leukemias and mantle cell lymphomas (Carmeliet and Jain, 2011). Notch induced cell cycle arrest in a variety of cell types, which could be reversed by mutations of Notch signaling (Sriuranpong et al., 2001). Notably, UXT is highly expressed in various tumor tissues, and serves as a co-activator of androgen receptor in prostate cancer. We have shown, in the context of angiogenesis, that deficiency of UXT blocks cell cycle at the G0-G1 stage, whereas DAPT treatment relieves cells from the blockage, demonstrating that UXT modulates the migration and division of endothelial cells through Notch signaling. It is intriguing for future studies to explore whether UXT promotes tumor metastasis by potentiating angiogenesis.

UXT is an α-class prefoldin family protein (Vainberg et al., 1998). The crystal structure of this protein is unknown. Bioinformatics analysis predicts that UXT harbors a large stretch of coil-coiled domain. Previously, we characterized UXT as a transcriptional co-factor for NF-κB, which plays important roles in inflammation and innate immunity. However, no investigation has addressed the *in vivo* function of UXT. We had failed to obtain UXT knockout mice, due to embryonic lethality. We are trying to generate a UXT conditional knockout mouse, so as to address whether UXT could modulate angiogenesis in mammals and, if so, whether UXT could attenuate Notch signaling in mice. Taken together, the present study uncovers UXT as a novel repressor of Notch signaling during angiogenesis, highlighting the yet-to-know essential functions of UXT in vertebrate development.

## MATERIALS AND METHODS

### Zebrafish maintenance and embryo production

Zebrafish maintenance, breeding and staging were performed as described previously (Kimmel et al., 1995). Wild-type (WT) AB, *Tg(kdrl:EGFP)^s843^* (Picker et al., 2002) and *Tg(fli1: nEGFP)^y7^* (Roman et al., 2002) zebrafish lines were obtained from Jiulin Du's (Institute of Neuroscience, Chinese Academy of Sciences, China) laboratory; *Tg(TP1:mCherry)* (Parsons et al., 2009) and *Tg(fli1: EGFP)^y1^* (Lawson and Weinstein, 2002) fish lines were obtained from the laboratory of F.L. The establishment and characterization of the transgenic lines has been described elsewhere (Chen et al., 2012; Yu et al., 2010).

### Riboprobe synthesis and whole-mount *in situ* hybridization

Antisense riboprobes for UXT were synthesized using the DIG RNA Labeling kit (Roche) according to the manufacturer's instructions. Whole-mount *in situ* hybridization was carried out as described previously (Gan et al., 2008; Lawson et al., 2001), and staining was performed with an alkaline phosphatase substrate kit (Promega).

### Western blotting

Embryos were deyolked as described previously (Link et al., 2006). Cell pellets were lysed with RIPA buffer (150 mM NaCl, 1 mM EDTA, 1% Triton X-100, 1% sodium deoxycholate, 0.1% SDS, 100 mM Tris-HCl pH 7.4) supplemented with protease inhibitor cocktail (Roche), and sonicated. Protein samples were resolved by SDS-PAGE, probed with anti-zebrafish UXT (ABmart, 4B4; 1:500) and anti-mouse GAPDH (ABmart; 1:1000), and visualized by enhanced chemiluminescence.

### Whole-embryo immunostaining of UXT

Embryos were fixed in 4% paraformaldehyde (PFA), and whole-embryo antibody staining of UXT was performed as described previously (Liu et al., 2003).

### *In vivo* confocal imaging and data analysis

*In vivo* confocal imaging was performed on the *Tg(kdrl:EGFP)^s843^* line at 28.5°C as described previously (Isogai et al., 2003). The length of ISVs and lumen diameters of dorsa aortas and posterior cardinal veins were calculated by using ImageJ analysis software (NIH). The calculation of the migration speed of tip cells was performed as described previously (Yu et al., 2010).

### Transplantation experiments

Transplantation was performed as described previously (Siekmann and Lawson, 2007; Yu et al., 2010). The donor *Tg(kdrl:EGFP)^s843^* was injected with 4 ng of control morpholino or 4 ng of UXT MO2; the *Tg(kdrl:EGFP)^s843^* was also injected with Rhodamine-561 dextran (Molecular Probes) as a lineage tracer at the one-cell stage.

### Real-time quantitative PCR (RT-qPCR)

Total RNAs were extracted from 50 zebrafish embryos using TRIzol reagent (Invitrogen) as described previously (Yue et al., 2009). All values were normalized to the level of GAPDH mRNA. The forward and reverse primers used are shown in supplementary material Table S2.

### Embryonic chromatin immunoprecipitation

E-ChIP was performed as described previously (Wardle et al., 2006; Yue et al., 2009). The E-ChIP primer sequences are provided in supplementary material Table S2.

### Reporter assays

Cells were seeded in 24-well plates and transfected with reporter gene plasmids combined with siRNAs and other constructs, as described previously (Sun et al., 2007).

### BrdU flow cytometry

BrdU flow cytometry was prepared following the APC BrdU Flow Kit (BD) manual instructions. HUVECs were transfected with shRNA or phage-UXT packaged in lentivirus. The concentration of DAPT or DMSO was 1.5 μM. HUVECs were stained with 1 mM BrdU (BD) for 2 h, and cytometric analysis was carried out on a BD FACScalibur Flow Cytometer.

### Co-immunoprecipitation and immunoblot analysis

Co-immunoprecipitaion was performed as described previously (Rustighi et al., 2009). See supplementary material methods for details.

### *In vitro* angiogenesis assay

The Matrigel assay (Arnaoutova and Kleinman, 2010) and 3D angiogenesis (Nehls and Drenckhahn, 1995) assay were performed as described previously. For details, please see supplementary material methods.

### DAPT treatment of zebrafish embryos

DAPT (Sigma) was dissolved in DMSO (300 μM stock solution) and added to system water with a final concentration of 60 μM. System water containing DMSO alone was used as a control. The embryos were dechorionated and added to system water containing DAPT or DMSO at 12 hpf. *In vivo* confocal images were taken at 30 hpf.

### Statistical analysis

Quantitative data are expressed as the mean±s.e.m. Statistical significance was determined by one-way analysis of variance. A *P*-value of less than 0.05 was considered statistically significant.

## Supplementary Material

Supplementary Material
