# Supplementary material for: UXT potentiates angiogenesis by attenuating Notch signaling
Source: Development. 2015 Feb 15;142(4):774–86. doi: 10.1242/dev.112532 (PMC4325377; doi:10.1242/dev.112532)
Supplement: Supplementary Material [file supp_142_4_774__index.html]

UXT potentiates angiogenesis by attenuating Notch signaling — Supplementary Material 

# UXT potentiates angiogenesis by attenuating Notch signaling

## DEV112532 Supplementary Material

**Files in this Data Supplement:**

- Supplementary Material
