# Supplementary material for: UXT potentiates angiogenesis by attenuating Notch signaling
Source: Development. 2015 Feb 15;142(4):774–86. doi: 10.1242/dev.112532 (PMC4325377; doi:10.1242/dev.112532)
Supplement: Supplementary Material [file supp_dev.112532_DEV112532supp.pdf]

Supplementary material methods

Cloning of Zebrafish UXT

Total RNA of 24hpf zebrafish embryos was extracted with Trizol Reagent (Invitrogen Life Technology), and reverse-transcription was using Promega Kit. The cDNA sequence of zebrafish UXT (ID: NM_001004671.1 Description: *Danio rerio* zgc:101894) was cloned by KOD-plus PCR, and primers used are listed in Table S2. The primers for cloning zf UXT-2M (I45P/L54P) are also provided in Table S2.

Morpholino, mRNA synthesis and micoinjection

Morpholino antisense oligonucleuotides (MO) were purchased from Gene Tools (Philomath, OR). UXT Morpholinos were designed as followed, UXT MO1 was targeted proper splicing of fifth exon of zebrafish UXT, and UXT MO2 was targeted to the start codon regions, and UXT MO3 was targeted to splicing of the third exon. The sequences of mophpolinos were listed in Table S1. To generate mRNAs, full-length UXT (zebrafish) gene was subcloned into pCDNA3.1 vector, and synthesized using the mMessage mMachine kit (Ambion). For all the microinjection experiments, stocks were diluted to different working concentrations and a volume of 1 nl was injected into one- to two-cell stage embryos.

Cell Culture

HEK293T and COS7 cells were cultured using DMEM (Invitrogen) plus 10% FBS (Invitrogen), supplemented with 1% penicillin-streptomycin (Invitrogen). Human

umbilical vein endothelial cells (HUVEC) were isolated from cord of newborns as described elsewhere (Baudin et al., 2007). HUVEC were cultured in M199 (Gibco) consisting of 20% Fetal Calf Serum (Gibco), 25 mM HEPES, 2 mM L-Glutamine, 1% penicillin-streptomycin (Invitrogen), 50 mg/ml Endothelial Cell Growth Supplement (BD) and 100mg/ml Heparin (Sigma).

Plasmids, siRNA Oligos, shRNAs and Transfection

Human UXT, UXT-2M (L50P/L59P) plasmids had been described previously (Sui et al., 2007; Sun et al., 2007).Human Notch-ICD, Notch-ICD truncations, RBP-Jκ-v4 cDNAs were amplified by PCR from human liver cDNA library and A549 cell cDNA, primers are listed in Table S2. The siRNA oligos against UXT has been described (Huang et al., 2011). The short hairpin RNA (shRNA) was designed targeting human UXT 5'-CAGCTGGCCAAATACCTTCAA-3' sequence. The negative control shRNA was targeting LacZ. Human UXT and UXT-2M cDNA sequences were cloned into pHAGE-flag vectors, and a vehicle vector was adapted as the control. Transient transfections of plasmids and siRNAs were performed with lipofectamine 2000 (Invitrogen) following manufacturer's instructions. shRNAs and pHAGE-flag were introduced into HEK293FT cells and packaged into lentiviruses (Invitrogen), and the titer of all lentiviruses were determined by multiplicity of infection (MOI) test.

Co-Immunoprecipitation and Immuno-blot Analysis

For transient transfection, HEK293T cells were cotransfected with indicated plasmids. 24 hr post-transfection, cells were harvested with lysis TBS buffer (50 mM Tris-Cl pH 7.4, 150 mM NaCl) supplemented with 1% NP-40, 1mM EDTA, 0.25% Sodium deoxycholate and protease inhibitors. After pre-clearing for 1 hour, lysates were incubated with the anti-flag agarose beads (Sigma) for two hours at 4°C. The beads were washed with TBS buffer containing 0.5% NP-40; then the beads were boiled using 1×SDS loading buffer for 5 min, and the supernatants were prepared for Western blot analysis. For endogenous IP experiemts, HUVECs were seeded in T75 Flask, after harvest, HUVECs were lysated by 0.5 mL HUVEC lysis buffer (50 mM Tris-Cl pH 7.4, 150 mM NaCl, 0.5% NP-40, 0.25% sodium deoxycholate, 2 mM sodium orthovanadate), mixed with antibody for Notch1 sc-6014 (Santa Cruz Biotechnology) overnight, added Protein G Agarose bead (Life Technology) for 4 hours, washed with TBS wash buffer.

***In vitro* Angiogenesis Assay**

HUVECs were transfected by shUXT or shNT and cultured in T25 for 3 days. For Matrigel tube formation assay, after harvesting, HUVECs were seeded on Matrigel (BD) in 96-well plates, then treated with 1.5 µM DAPT or DMSO. *In vitro* angiogenesis assay was performed essentially as previously described (Arnaoutova and Kleinman, 2010). HUVECs were finally stained with Calcein AM (Invitrogen), and the images were obtained by fluorescence microscopy (Olympus). For three-

dimensional Angiogenesis assay, cytodex microcarriers (GE) were pretreated and coated with HUVECs as described (Nehls and Drenckhahn, 1995), and were embedded in the collagen gels (Invitrogen). After polymerization (30 min) in 96 well plate, 100 μl of culture medium (M199 with 20% FBS, L-Glutamine, 25 mM HEPES, 50mg/ml ECGS, 2 mM 100 mg/ml Heparin) with 40 ng/ml VEGF and 40 ng/ml FGF-b were added into each well. After 24 hours, *in vitro* angiogenesis was quantified.

Notch1 Stability and Cleavage Assay

HUVECs were transfected either with empty vector, or shUXT or phage-UXT-Flag by lentivirus infection. For Cycloheximide (CHX) pulse experiments, cells were treated with DMSO or CHX (20 μM) for indicated time and the whole cell lysates were harvested and probed by anti Notch1 antibody (Santa Cruz). For cleavage assay, after transfection, HUVECs were harvested and probed by anti Notch val 1744 antibody (Cell Signaling).

Table S1. Sequences of Morpholino Oligonucleuotides

Morpholino	Sequence (5' to 3')
UXT MO1	CACTACTAAAATCCTCTTACTCTGT
UXT MO2	ATAACAGATGTGCCAGAAGTCATGA
UXT MO3	CTTTCCTGAAGGTGAATCAAACACA
RBP-Jκ MO	CAAACTTCCCTGTCACAACAGGCGC(Siekmann and Lawson, 2007)
Dll4 MO	CTTTGGGTGGCCGGTAAGCTCGAAC(Siekmann and Lawson, 2007)
Notch1b	AATCTCAAACTGACCTCAAACCGAC (Milan et al., 2006)
Control MO	CCTCTTACCTCAGTTACA ATTTATA

Table S2. Primer list for RT-PCR, E-ChIP and Cloning

RT PCR	Forward 5' to 3'	Reverse 5' to 3'
zf cox2	ATGAATAAACTGGTTTGCTTGGTC	TCCTGGTGCAGTCACATTCATAA
zf nestin1	AAGACGTCCAACCTGTTTCTGATA	CAGGAGAACATGGAAACCATGC
zf angpt-1	ATGTGGTGGGGTTGCTTGTT	TCCATGCTGGATCCGGTGA
zf flt1	ATGATATTTGGACTGTCAGGACG	AACCCACTGGAGCTCCCA
zf KDR	AACATCCTCCTCACCTTCAACGT	AGGTGTGGACCACTCCAGAATT
zf hey1	ATGAAGAGAAATCACGATTT	TGGAGACATTGACCCGAGTG
zf her6	ACATCATTGCCGCACCAGTT	CATGATATCGGCAGGCATCT
zf rbpja	CAGTGCTCATACTGCATGCA	CGAACAGCCTTCTCTCTCCA
zf rbpjb	TTGTGACAGGGAAGTTTGGG	TACACACGGGGGAGGACAGA
zf notch1a	ATGAACCGTTTCTTGGTGAA	GCTGGGCTTGTACTCGCATA
zf notch1b	ATGCATCTTTTCTTCGTGAA	TCTCCCGTCTGCAGTTGGTT
zf gapdh	CCAACTGCCTGGCTCCTT	CCCATCAACGGTCTTCTGTG
zf dll4	ATGGCAGCTTGGCTCACCTT	TGCGAGCAAGCCTTTGAAAT
zf uxt	GCTCTGCCAGGAAAGTGCTG	CACATACCTGAATGCTTTG
zf vegfaa	TGCTCCTGCAAATTCACACAA	ATCTTGGCTTTTCACATCTGCAA
zf vegfab	ATAACAAGTCTCTCTGCCGT	ATGAAAATGTCACCCTGATG

zf heyl	ATCATTGAGCACCCAGGGGA	TCAAAACGCCCCTATCTCTGT
zf hey2	ATGAAGCGGCCCTGTGAGG	CTCAAAAGCTGTTGGCACCAGA
human hes1	ATGCCAGCTGATATAATGGA	AGGCTTTGATGACTTTCTGT
human hey1	AAACTGTCGCCGCCTCT	TTAAAAAGCTCCGATCTCC
human heyl	ACAGCTACGCAGCCGAGATG	CAGGATGATGCCTGTGGCTC
human hey2	GGATTCAGCCCTCCGAATGC	ACTGCTGCTGCTGCGTTTGG
**E-ChIP**	**Forward 5' to 3'**	**Reverse 5' to 3'**
Hey1	ACGCTCAGACACGCCTCTCT	CGTATCCTCAGCGTGCGTCA
Her6	CCTCGGGCCGCGTGTCC	ATATCTCCGGCTGCACGCGA
Her9	CTGAAGCGAGTGTGGGAAAGA	AGTCGAATCTCCCACTGCCA
Cdx4	CGTCGATCTGGCATCAGTCT	TCGGGCGAGCAATAGGAA
GAPDH	CAGAGGCTTCTCACAAACGG	AGGCTTCACCAGCGCAAT
**Cloning**	**Forward 5' to 3'**	**Reverse 5' to 3'**
zf uxt	TATAAGCTTATGACTTCTGGCACATCTG	TTTCTCGAGCTAAAACACCTCTCTCCT
homo NICD	GTCCCGCAAGCGCCGGCGGCAGCAT	TTACTTGAAGGCCTCCGGAATGCGG
homoRBP-Jk□	ATCGGATCCATGGCGTGGATTAAAAGGAAATTTG	ATCTCTGAGTTAGGATACCACTGTGGCTGTAGAT
ΔRAM (phage-flag)	ATCTCTAGAATGCCAGACAGACCGCACGGGCGAG	ATCGGATCCTTACTTGAAGGCCTCCGGA
ΔTAD-Flag	CAAGGAGCAGCCACTGGGCCCCAGCAGCCTGG	GGCTGCTCCTTGCTTCCACAGGCCAGGCCTTT
ΔANK-Flag	GCACAAGGCCAAGGACCTCAAGGCACGGAGGA	TTGGCCTTGTGCAGGCTGGCGCCCTGGTAGAT
ΔPEST-Flag	CGTTAACTCGAGGGGGGGCCCGGTACCTT	CTCGAGTTAACGTCTGCCTGGCTCGGCTCTCC
zfUXT(L45P)	TATGAGAAAATCGCACAATACCCGCA GCTGAAGAA	TGCTTTGTATTGTGTTCTTCAGCTGCGGGTATTGT
zfUXT(L54P)	GAAGAACACAATACAAAGCCCCCAGGAAAGCGGCA	TTTTCAGTTCTTTGCTGCCGCTTTCCTGGGGGCTT

Supplementary material references

Arnaoutova, I. and Kleinman, H. K. (2010). In vitro angiogenesis: endothelial cell tube formation on gelled basement membrane extract. *Nat Protoc* **5**, 628-35.

Baudin, B., Bruneel, A., Bosselut, N. and Vaubourdolle, M. (2007). A protocol for isolation and culture of human umbilical vein endothelial cells. *Nat Protoc* **2**, 481-5.

Huang, Y. F., Chen, L. A., Zhou, Y., Liu, H., Yang, J. Q., Liu, Z. G. and Wang, C. (2011). UXT-V1 protects cells against TNF-induced apoptosis through modulating complex II formation. *Molecular Biology of the Cell* **22**, 1389-1397.

Milan, D. J., Giokas, A. C., Serluca, F. C., Peterson, R. T. and MacRae, C. A. (2006). Notch1b and neuregulin are required for specification of central cardiac conduction tissue. *Development* **133**, 1125-32.

Nehls, V. and Drenckhahn, D. (1995). A novel, microcarrier-based in vitro assay for rapid and reliable quantification of three-dimensional cell migration and angiogenesis. *Microvasc Res* **50**, 311-22.

Siekmann, A. F. and Lawson, N. D. (2007). Notch signalling limits angiogenic cell behaviour in developing zebrafish arteries. *Nature* **445**, 781-4.

Sui, S. G., Tang, Y. J., Lou, X. W., Zhu, L. H., Yang, K., Zhang, B. H., Shi, H. X. and Wang, C. (2007). UXT is a novel and essential cofactor in the NF-kappa B transcriptional enhanceosome. *Journal of Cell Biology* **178**, 231-244.

Sun, S., Tang, Y., Lou, X., Zhu, L., Yang, K., Zhang, B., Shi, H. and Wang, C. (2007). UXT is a novel and essential cofactor in the NF-kappaB transcriptional enhanceosome. *J Cell Biol* **178**, 231-44.

Supplementary Figures

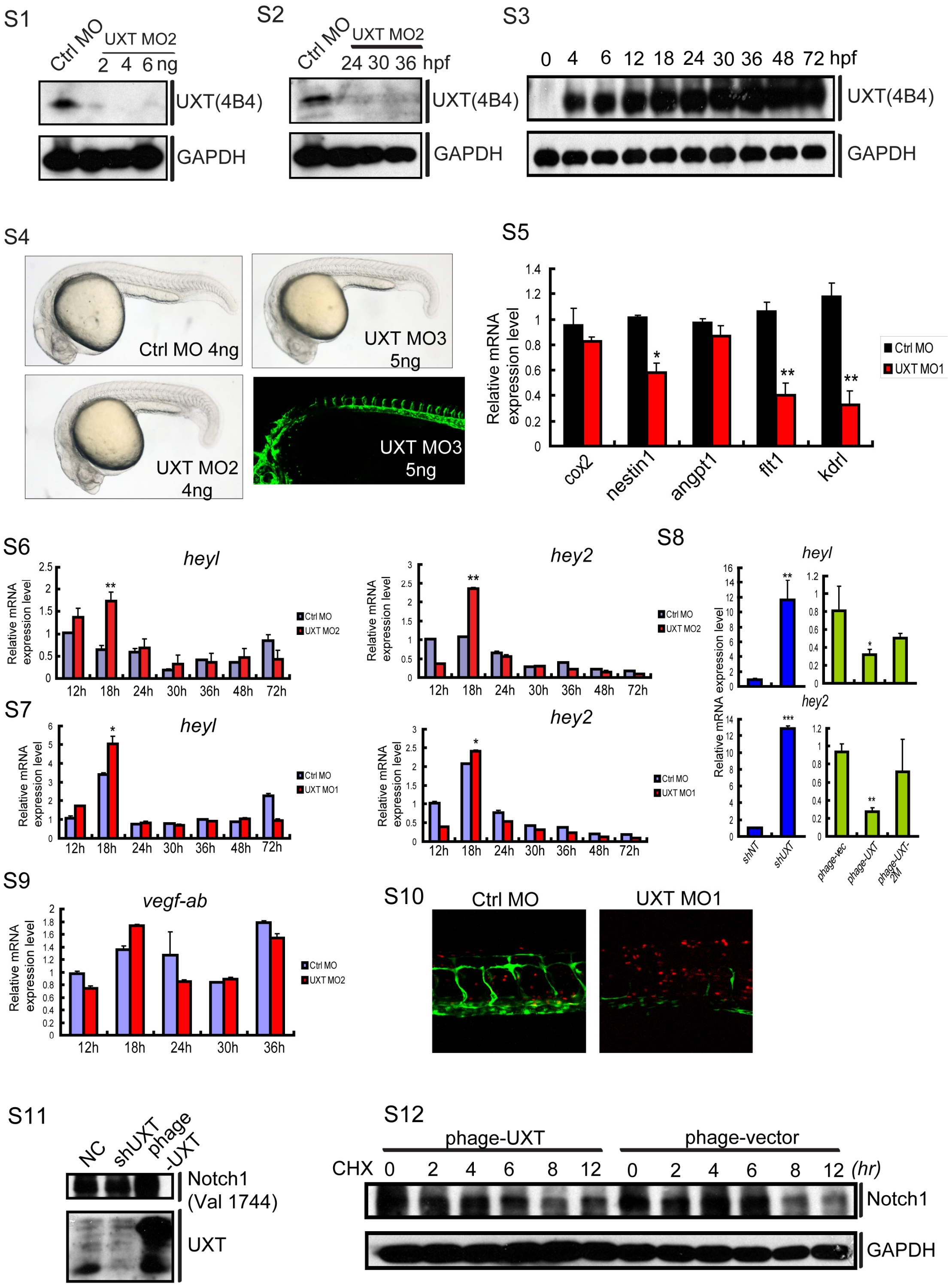

Fig. S1. The antibody staining of UXT in zebrafish embryos injected with control MO, 2 ng, 4 ng or 6 ng UXT MO2 at 24 hpf were analyzed.

S2. The antibody staining of UXT in zebrafish embryos injected with control MO, 4 ng UXT MO2 at 24 hpf, 30 hpf or 36 hpf respectively were analyzed.

S3. The antibody staining of UXT in wild type zebrafish embryos at the time window from 0 hpf to 72 hpf were analyzed.

S4. Phenotypic analyses of zebrafish embryos at 24 hpf. Embryos were injected with 4 ng control morpholino (Ctrl MO), 4 ng UXT MO2, or 5 ng UXT MO3 and the confocal Image of *Tg(kdrl:EGFP)*^{s843} embryos injected with 5 ng UXT MO3 were analyzed.

Fig. S5. Real-time quantitative PCR (RT-PCR) verification of angiogenesis markers in 30 hpf zebrafish embryos injected with Ctrl MO or with 5 ng UXT MO1. Data shown are mean±SEM of at least three independent experiments,*p<0.05, **p<0.01, ***p<0.001 versus the corresponding control.

Fig. S6.UXT attenuates the expression of Notch target genes. The control and UXT-deficient zebrafish embryos (from 12 hpf to 72 hpf) were harvested, and the expression of *heyl*, and *hey2* were measured by Real-time quantitative PCR. Data shown were mean±SEM of at least three independent experiments,*p<0.05, **p<0.01, ***p<0.001 versus the corresponding control.

Fig. S7. The Real-time quantitative PCR verification of *heyl* and *hey2* in zebrafish embryos injected with 5 ng UXT MO1. Data shown were mean±SEM of at least three independent experiments,*p<0.05, **p<0.01, ***p<0.001 versus the corresponding control.

Fig. S8. UXT directly attenuates Notch targeted genes in endothelial cells. The relative expression level of *heyl* and *hey2* were measured by Real-time PCR. The data were normalized on the basis of the corresponding input control, and presented as mean±SEM of at least three independent experiments,*p<0.05, **p<0.01, ***p<0.001 versus the corresponding control.

Fig. S9. The relative mRNA expression level of *vegfab* in control and UXT deficient embryos (from 12 hpf to 36 hpf). Data shown are mean±SEM of at least three independent experiments,*p<0.05, **p<0.01, ***p<0.001 versus the corresponding control.

Fig. S10. UXT attenuates Notch signaling *in vivo*. Confocal Images of *Tg(TP1:mCherry; fli1: EGFP)*^{y1} Fish injected with Ctrl MO or 5 ng UXT MO1.

Fig. S11. UXT does not affect the cleavage of Notch1. HUVECs were treated with shUXT or phage-UXT-Flag. The endogenous NICD were immunoprecipitated by anti-Notch1 val 1744 antibody.

Fig. S12. UXT does not affect the stability of Notch1. HUVECs ectopic-expressed with UXT or empty vectors were treated with 20 mM CHX for the indicated time. The cell lysates were probed by anti-Notch1 antibody.
